# Death Rates of Elite and Professional American Athletes During Early Adulthood are Lower than the General Population

**DOI:** 10.1177/15598276261418585

**Published:** 2026-01-26

**Authors:** John W. Orchard, Kimberly G. Harmon, Nicholas D. Orchard, Cindy J. Chang, Danica Sardelich, Jessica J. Orchard

**Affiliations:** 1 School of Public Health, University of Sydney, Sydney, Australia (JWO, JJO); 2 Department of Family Medicine and Center for Sports Cardiology, University of Washington, Seattle, WA, USA (KH); 3 179991 Sydney Grammar School, Sydney, Australia (NO); 4 Department of Orthopedics, University of California, San Francisco, CA, USA (CJC); 5 Sports Clinic, University of Sydney, Sydney, Australia (DS)

**Keywords:** safety, mortality, elite athletes, deaths in sport

## Abstract

This study aimed to assess rates of death of elite and professional young adult athletes in different US sports, comparing to the general population. Male and female athletes’ birth and death dates were analyzed to compare rates of death from ages 21–40 years inclusive between the athlete groups and the general population. Data were downloaded from Wikidata for notable US athletes, and Standardized Mortality Ratios (SMRs) were calculated against mortality rates by year (1950–2022 inclusive) for the USA general population by age and sex. Sports were included based on sufficient cohort size for analysis. Results were obtained for 54 648 male and 6280 female notable athletes from 17 sports. Overall SMRs (95% confidence intervals) for athletes were 0.49 (0.46–0.53) for males and 0.38 (0.26–0.55) for females. The majority of sports, including football, baseball, basketball, hockey, tennis, and golf in males, and track and field, soccer, and rowing in both males and females, had significantly lower mortality rates in athletes than the general population. The only sports with significantly higher mortality rates in male athletes were auto racing, mountaineering, and professional wrestling. For the majority of major sports, professional and elite athletes have lower death rates than the general population in early adulthood.

“For the majority of sports, professional and elite athletes have lower death rates than the general population in early adulthood.”

## Introduction

Elite athlete cohorts in most sports outlive the general population,^1-3^ although there are some exceptions.^4,5^ By population, the USA is the third largest country in the world, and with such a diverse sporting culture it has the largest cohort of elite and professional athletes. Many USA athlete cohorts have been studied assessing lifelong death rates compared to the general population (with a focus on middle-age onwards, when most deaths occur). American football players,^6^ baseball players,^7^ basketball players,^8^ and Olympic sport athletes from multiple sports (both male and female)^9^ all tend to outlive the general population. Within sports, certain causes of death (such as neurodegenerative deaths within soccer or American-style football players) can exceed the expected rate for the general population,^10^ but this does not necessarily result in an overall higher mortality rates because of reduced risk of other diseases.^11^ One published study compared active professional athletes in the four major leagues in the USA (football, baseball, basketball, and hockey) and found that they tended to die from similar causes to the general population, but did not calculate Standardized Mortality Rates (SMRs) compared to the general population.^12^

There have also been multiple publications which focus on annual death rates of college-age athletes.^13-15^ With the exception of specific causes in specific groups (for example, sudden cardiac deaths in black male Division I basketball players),^16^ college-age athletes also have relatively low death rates compared to the general population. To our knowledge, there have not previously been studies which have focused on death rates of US athletes in early adulthood beyond the college years.

The ability to assess death rates of athletic populations has improved in recent years as databases like Wikipedia, Wikidata, and athlete almanacs hold publicly available records of births and deaths of major athletes.^12,17^ The data contained within these sets is highly accurate (particularly for English-language notable people) and extensive.^18^ This study set out to assess the overall risk of death for notable US athletes in the early adulthood years (ages 21–40 inclusive), comparing sports to each other and the general population, using these publicly available records. This is an age cohort which has not been previously studied to our knowledge. In addition, this study will examine one of the largest cohorts to date of female athletes to compare to the general population.

## Methods

We aimed to calculate Standardized Mortality Ratios (SMRs) of American (USA) athletes in early adulthood (ages 21–40) from multiple different sports where there were sufficiently large cohorts of publicly listed notable athletes.

### Inclusion Criteria

#### Chronological Ages

We included athletes from the age of 21 (entry on 21^st^ birthday), as this is generally the earliest age at which athletes would have left the college sporting system and also is one of the conventional starting ages of adulthood. Exclusion from the cohort was upon reaching the age of 41 or death (prior to age 41). The maximum age of 40 was chosen to allow 20 years of follow-up in early adulthood for the majority of the cohort and also as this age would represent one by which the vast majority of athletes would have retired from professional/elite sport.

#### Years of Surveillance

We enrolled athletes from the start of the year 1950 until the end of the year 2022. 1950 was chosen as a starting year as it was sufficiently after the Second World War (which would have acted as a major confounder for deaths of young adults) and at a time after which professional sport had become well established in the USA. The year 2022 was the final year which had both age standardized death rates for USA registered in the Human Mortality Database (as at 2024) and sufficient lag time for public recording of deaths to have been made.

#### Sport and Athlete Cohorts

Athletes were defined (included) according to whether they had a personal entry on Wikidata. The Wikipedia definition of a notable athlete (that is, one who warrants a Wikipedia page entry and whose page would not be taken down if created) is “an athlete is likely to have received significant coverage in multiple secondary sources, and thus be notable, if they have been successful in a major competition or won a significant honor.”^19^ Sports were chosen from popular sports where a sport/sex combination had at least 250 notable athletes from that sport who had reached the age of 41 years or 10 or more notable athletes who had died before the age of 41. These values were chosen in order to minimize insignificant results (i.e., with either numerator or denominator large enough to have a good chance of generating a significant result for Standardized Mortality Ratio). We combined all track and field sports into a single category, all aquatic sports (swimming, diving, and water polo) and alpine skiing, figure skating, and snowboarding into a winter sports category.

To be included, the athlete’s nationality also needed to be registered as USA (either exclusively or as a dual national including USA). They also needed a tag for the sport in question and for that sport to be referred to in some way in their “Person description” (in order to exclude celebrities such as team owners, musicians, or politicians who had a sport tag on Wikidata but were notable persons outside that sport). The size of each athlete cohort was determined by the number of notable athletes on Wikidata.

A very small number of athletes were considered to be professional athletes in more than one sport. For simplicity, these athletes were categorized only as playing a single sport, being the one that they had the most seasons playing as a professional from the years 21–40 inclusive.

### Downloading of Athlete Data

The SPARQL script in Appendix 1 was used to obtain player data from Wikidata.

All primary downloads were done in July 2024.

The scripts were saved as .csv files, and then imported into Excel (Microsoft, Seattle). For each sport, a separate Date of Birth (DOB), Date of Death (DOD), and Player Description .csv file was downloaded. These were then imported into an Access database (Microsoft, Seattle) to merge the DOB and DOD files and to flag and remove duplicates from the analysis.

### Removal of Duplicates

Where a player had two dates of birth supplied on Wikidata, if they were both in the same year we just chose the later date and deleted the earlier one as a duplicate. For the majority of athletes who lived beyond the age of 40, year of birth was a sufficient identifier. If two years of birth were recorded, we used Wikipedia as the primary adjudicator to determine actual years of birth.

For athletes who had died at either age 20 or age 21, or age 40 or age 41, we required exact date of birth and exact date of death. A death prior to the 21^st^ birthday meant that the athlete was never recruited into our cohort, whereas a death on the 41^st^ birthday or beyond meant that an athlete was considered to have survived beyond the age of 40.

Finally, any players who had identical first name and surname to other famous players (particularly fathers and sons who both played the same sport with the same first and last name) were individually checked to make sure a young death was not assumed erroneously (for example, by the date of birth of the son being erroneously paired with the date of death of the father with the same name).

### Categorization of Deaths

For those sports which were revealed to have an excess of deaths of athletes between the ages of 21 and 40 inclusive, the deaths were categorized, where possible (that is, if cause was listed on Wikipedia), as occurring either during sports participation or outside of sports participation.

### Statistical Analysis of Expected Compared to Actual Deaths

The number of included athletes in the cohort for each year from 1950 to 2022 was then calculated. Death rates (expected deaths) for the athlete populations were calculated as if the make-up of the general population aged 21–40 was proportionate to the comparison athlete population by age, sex and year over the 72-year time period. This was done on an annualized basis using single year age-group cohorts. For example, in a sport with 141 male athletes who were aged 36 in 2019, with the Human Mortality Database indicating a death rate in males of 0.002202 for 36yo US males in 2019, then the expected number of deaths for those 141 athletes in 2019 was 0.3104 (141 multiplied by 0.002202). For the sport as a whole, the expected number of male deaths for the entire cohort in a given year was the sum of the 20 individual age cohorts (21 through 40).

Calculation of SMR was by the rate of actual (observed) deaths divided by expected deaths. Confidence intervals at 95% level for the SMRs were calculated using Poisson distributions for observed death counts <100 and the normal distribution formula SMR ±1.96 * (SQRT(Actual deaths)/Expected deaths) for observed death counts >100.^20^

For players who had died in sports which had an excess number of observed deaths, we referred to, in order, cause of death on Wikidata, description of death on Wikipedia followed by Google searches, to be able to characterize deaths as being related to their sport or not.

### Ethical Considerations

All of the data examined was publicly available with the data presentation being of large cohorts. We used the online NIH tool at https://grants.nih.gov/policy-and-compliance/policy-topics/human-subjects/hs-decision to determine that for all three reasons of (1) only using publicly available data; (2) identification of deceased athletes no longer has privacy risks; and (3) presentation of data is of pooled risks rather than individual birth and death dates, that this project did not require Ethics Committee oversight. Our local ethics committee also confirmed in writing that studies using only publicly available data do not require evaluation.

## Results

### Males

The following sports reached sufficient cohort sizes for male athletes for inclusion: Aquatic sports (swimming, water polo, and diving), Winter sports (skiing, snowboarding, and figure skating), American-style football, Auto racing, Baseball, Basketball, Boxing, Equestrian sports (including jockeys), Golf, (Ice) Hockey, Mixed martial arts, Mountaineering, Professional wrestling, Rowing, Soccer, Tennis, and Track and Field. All of these sports qualified by having at least 250 notable participants of relevant age on the Wikidata project except for male Mountaineers, which only had 116 notable participants but who had recorded 11 deaths between ages 21 and 40.

### Females

The following sports reached sufficient cohort sizes for female athletes for inclusion: Aquatic sports (swimming, water polo, and diving), Winter sports (skiing, snowboarding, and figure skating), Basketball, Golf, Rowing, Soccer, Tennis, and Track and Field.

Notable sports which did not qualify for the analysis due to lack of notable athlete numbers include cycling, lacrosse, powerlifting, Gymnastics (artistic), Olympic wrestling, and sailing.

Table 1 shows the number of participants for each sport and raw death rates (percentage of athletes who died between 21 and 40). Table 2 shows the calculated SMRs against the USA general population by sex and age for the profile of athletes in each sport.

**Table 1.** Cohort Numbers and Raw Death Rates Between Ages of 21 and 40 by Sport.

Sport	Male athletes	Female athletes^a^	Male raw death rate	Female raw death rate
American-style football	24 734		1.52%	
Aquatic sports	601	542	1.50%	0.55%
Auto racing	996		7.33%	
Baseball	3403		1.15%	
Basketball	8319	805	1.56%	0.62%
Boxing	812		3.57%	
Equestrian	250		1.20%	
Golf	1210	335	0.74%	0.90%
Ice hockey	2782		0.83%	
Mixed martial arts	1071		1.03%	
Mountaineering	116		9.48%	
Professional wrestling	1373		5.39%	
Rowing	833	430	0.60%	0.23%
Soccer	4787	1709	0.33%	0.12%
Tennis	714	691	1.12%	0.58%
Track and field	1985	1113	1.26%	0.54%
Winter sports	662	655	2.72%	0.76%
ALL INCLUDED SPORTS	54 648	6280	1.57%	0.46%

^a^Blank entries indicate that sample size for females in the relevant sport was insufficient for inclusion.

**Table 2.** Standardized Mortality Rate by Sport (Compared to USA General Population) Between Ages 21 and 40.

Sport	Male deaths (21–40 years old)			Female deaths (21–40 years old)^a^		
	Expected	Actual	SMR (95% CI)	Expected	Actual	SMR (95% CI)
American-style football	796.3	376	0.47 (0.42–0.52)			
Aquatic sports	19.2	9	0.47 (0.21–0.89)	7.2	3	0.42 (0.09–1.21)
Auto racing	32.3	73	2.26 (1.77–2.84)			
Baseball	115.1	39	0.34 (0.24–0.46)			
Basketball	269.5	130	0.48 (0.40–0.57)	10.8	5	0.46 (0.15–1.08)
Boxing	27.3	29	1.06 (0.71–1.52)			
Equestrian	9.3	3	0.32 (0.07–0.95)			
Golf	42.6	9	0.21 (0.10–0.40)	5.4	3	0.55 (0.11–1.62)
Ice hockey	83.4	23	0.29 (0.17–0.41)			
Mixed martial arts	31.7	11	0.35 (0.17–0.62)			
Mountaineering	4.2	11	2.60 (1.30–4.66)			
Professional wrestling	47.1	74	1.57 (1.24–1.97)			
Rowing	29.3	5	0.17 (0.06–0.40)	6.1	1	0.16 (0.09–0.91)
Soccer	125.7	16	0.13 (0.07–0.21)	13.4	2	0.15 (0.02–0.54)
Tennis	24.0	8	0.33 (0.14–0.66)	9.4	4	0.43 (0.12–1.09)
Track and field	64.8	25	0.39 (0.25–0.59)	14.5	6	0.41 (0.15–0.90)
Winter sports	19.6	18	0.92 (0.54–1.45)	8.5	5	0.59 (0.19–1.38)
All sports	1742.0	859	0.49 (0.46–0.53)	75.4	29	0.38 (0.26–0.55)

^a^Blank entries indicate that sample size for females in the relevant sport was insufficient for inclusion.

For the sports with a raw excess number of deaths: at least 5 of 29 male boxers died during boxing bouts; at least 27 of 73 male auto racing competitors died behind the wheel (in races or training); and 10 of 11 male mountaineers died while climbing. However, we were unable to ascertain whether any of the 74 male professional wrestlers died during competition.

## Discussion

This study examines the relative safety—in terms of risk of early death—of being a participant in the major US sports in early adulthood (ages 21–40) at elite/professional level. The overall SMR of both male and female athletes (for all sports combined) was substantially lower than the general US population of the same sex at the same ages. Most sports followed similar patterns with only a few exceptions.

### The “Big 5” Male Spectator Sports

The largest cohorts were for male athletes in the so-called “Big 5” sports of American-style football, baseball, basketball, hockey, and soccer. Young athletes in all these sports had a substantially lower risk of death compared to what would be expected based on general population mortality data. Baseball, soccer, and hockey had particularly low SMRs in this age group.

American-style football had a significantly lower overall SMR than the general US male population. Although American football has faced media criticism in recent years regarding player safety, neither heat-related deaths,^21^ cardiac, neurological deaths, nor any other cause^22^ led to any excess death rates in early adulthood in footballers. An overall conclusion is that notable male American football players die at rates lower than the general American male population in early adulthood.

Basketball also had a significantly lower SMR than the general US male population in this age group. Sudden cardiac deaths have been shown in college-age basketballers to be higher than expected in the general student population.^16^ However, in our cohort of post-college-age players, male basketball players outlived the general American male population of the same age. It is possible that there might be data limitations, but also possible that this finding reflects that cardiac conditions leading to premature death in basketballers are to a large extent congenital^16^ and more likely to affect college-age players than adults after the age of 21.

Baseball, soccer, and hockey had a very reassuring safety profile between the ages of 21 and 40 in terms of lack of premature deaths.

### Sports with Higher Rates of Early Adulthood Deaths in Male Athletes

Mountaineering and auto racing had higher rates of death in early adulthood that related directly to participation in the sport, with multiple deaths reported during the specific sport in question. The sample size for mountaineering was low, so there is less confidence about the increased magnitude of risk from our cohort. However, other publications which draw upon larger cohorts of mountaineers from multiple countries confirm that this is indeed one of the riskiest sports for participant death.^23-25^ Including non-elite athletes, it is estimated that an Everest summit ascent has an absolute risk of death of 1 in 100, and there are other mountains with even higher absolute risk of death than this.^26^

Auto racing also has a high risk of death in early adulthood and it can be concluded from this cohort that participants are at greater risk of death than members of the general US population. Although this has been accepted within the industry, it is reassuring that safety improvements have been implemented in recent years, which is likely to be lowering the risk of death.^27^

Professional wrestling has a high risk of death for male competitors in young adulthood, but it is uncertain whether this directly relates to sports participation. Previous study has also found a high rate of premature death among pro wrestlers, due to cardiac disease and other causes possibly indirectly related to the sport.^5^

Boxing is a sport with a significant risk of death “in the ring”^28^ but with an overall risk of death in our cohort that was similar to the general population of the same age. The fact that 5 of 812 notable male boxing participants died from injuries sustained during boxing matches is concerning. However, the overall expected number of deaths during early adulthood (age 21–40) for male participants in this sport from all causes was 27, and as 29 were observed this did not equate to a significantly increased rate of premature deaths overall.

### Sports with Lower Risk of Death for Male Athletes in Early Adulthood

Aquatic sports (swimming, water polo, and diving), equestrian sports, golf, mixed martial arts, rowing, tennis, and Track and Field all showed healthy safety profiles among our young US male cohorts.

### The Safety of Female Elite and Professional Athletes

All of the studied female sports had lower SMRs than the general US population of the same age. Sample size allows us to be particularly confident about the safety of basketball, aquatic sports, rowing, soccer, tennis, and track and field for female athletes in early adulthood. Basketball is known to have a lower risk of sudden cardiac death in young females than young males.^16^ Of the female sport cohorts, neurodegenerative concerns later in life probably only relate to the sport of soccer, with no evidence of premature deaths in female soccer players being observed in the years studied. Sample size did not allow us to assess mountaineering, auto racing, the collision football codes, equestrian sports, or professional wrestling in females, but we would possibly expect similar concerns in females to males participating in these sports. As this is one of the first studies looking at multiple female sports, further study of females is warranted in the future.

For the general US population between the ages of 21 and 40, males are far more likely to die than females, particularly related to gun violence, automobile accidents, and drug overdoses,^29^ illustrated in our study by Tables 1 and 2. The best illustration would be winter sports, where the male and female sample size is very similar (Table 1), yet there are approximately double the number of “expected” deaths among the male group (Table 2), indicating that US males are about twice as likely as US females to die in this age group in the general population.

### Cohort with Limited Sample Size and Anomalous Death Rates

There were multiple sports in females with low SMRs but where the 95% confidence intervals included 1.0 indicating that the lower death rates did not reach statistical significance, including aquatic sports, golf, basketball, and tennis. For most of these sports, the primary explanation is likely to be small sample size. When combined, the sample size allows us to be more confident about the safety of female athletes in early adulthood.

The winter sports as a combined group, for both males and females, exhibited an only marginally reduced risk of death, without reaching statistical significance. However, we noted that many of the deaths related to the tragedy of Sabena Flight 548, where 18 figure skaters were killed (along with all other occupants on board) in 1961. Our current study (with a finish year of 2022) did not capture the remarkably similar tragedy of American Airlines Flight 5342 in January 2025 which also took the lives of a substantial number of notable figure skaters. It is likely that athletes (of both sexes and in all sports) fly far more often than the general population and hence may be more at risk of death in plane crashes. However it is probably true that athletes, like the general population, are much more likely to be killed in motor vehicle collisions than plane crashes.^13^

### Strengths and Limitations of Our Study Design

The major strength of this study was the use of open access data (from Wikidata) using methods that could be replicated by other researchers. Results would vary only slightly if different decisions were made about inclusion or exclusion of certain individuals or sports. Handling of duplicates should be the same if the research was repeated, but there is room for small error or judgment calls in data curation, both in the dataset at Wikidata and at the analysis end. Our results appear to be consistent with individual sport-by-sport analyses performed by other smaller cohort research studies, making it more likely that our characterizations of each sport are accurate.

The major limitation of this study is potential selection bias for what constitutes a “notable” athlete on the Wikidata project and whether this might over- or under-estimate the death rates in the sports we analyzed. This potential selection bias would be lower for sports with a very large sample size of notable athletes (e.g., male American-style football players, baseball and basketball and hockey players). The risk of bias might be higher for sports with smaller sample sizes. The most important strength is that athletes in this category of being “notable” will almost certainly receive publicity if they died at a young age (less than 40). Although it is a potential limitation that birth and death data may be potentially inaccurate on Wikipedia and Wikidata due to open-source editing, there is some research to confirm that their accuracy is excellent,^18,30,31^ likely to be because errors are quickly corrected by other users.

To obtain consistency across sports, we chose age 21 and age 40 as start and finish of the observation period rather than starting at the time of professional “debut,” because of inconsistency in ages at which athletes start professional/elite sport. This may have introduced a selection bias in that some athletes may not have been notable by the age of 21, and if they “became” notable in their mid-late 20s then they of course could not have previously died prior to becoming notable. For the major sports, achieving a major league contract (e.g., NFL, NBA) seems to automatically constitute notability and in the vast majority of circumstances this would occur in early 20s.

Selection bias in terms of conferring notability with regards to death may occur in both directions. For example, an unusual death during sports participation may confer posthumous notability for a second-tier athlete and this bias would potentially overestimate the death rate of notable athletes. An athlete killed early in their career (say at age 21) from another cause (say motor vehicle accident) might never have had the chance to become “notable” by reaching a high level of achievement in the sport.

In terms of cutoffs we used to decide whether to include a sport/sex group, the minimum sample size of notable athletes (250) allowed a large number of sports to be considered, with most revealing significant results. The second qualification of 10 or more deaths allowed an extra group (male mountaineers) to also be assessed, and a significant result was found in this group as well. If the cutoff required 10 or more deaths to be necessary to assess any sport, none of the female sport groups would have qualified for inclusion, as none of them had 10 or more deaths in any athlete group.

All sports are also subject to the Health Worker Hire Effect (HWHE) whereby the athletes will generally be free from chronic health conditions at the start of their athletic career, and would hence be expected to outlive the general population. Although the general population is a good universal comparator, most athletes should be expected to outlive this cohort by a certain amount. Athletes have been found to have lower death rates than rock musicians,^32^ a comparable (in terms of early-life fame) group of notable people who unlike athletes tend to die prematurely at higher rates than the general population.^33,34^

Finally, we did not sub-divide athletes by any other feature than sex, and in particular not by socioeconomic status or race. This would be difficult as mixed race athletes are common and socioeconomic class (prior to starting as an elite athlete) is difficult information to obtain accurately and changes throughout an athlete’s career. It is possible that socioeconomic background might (also) bias some of the results, if, for example, certain sports such as baseball and rowing attracted athletes from a higher socioeconomic background than other sports such as boxing and basketball.

Although we cannot directly comment on athletes of lower levels, based on the known protective effects of physical activity on all-cause mortality,^35,36^ we would hypothesize that athletes at all levels (below professional) also would probably outlive the general population.

## Conclusion

Most of the major sports have a healthy safety profile in early adulthood as athletes outlive the general population between the ages of 21 and 40. This applies to the Big 5 male professional sports, all studied female sports, and most Olympic sports.

The sports with the most concerning safety profiles include mountaineering and auto racing (risk of death while participating in a dangerous environment) and professional wrestling (albeit not determined whether this is sport related or lifestyle related).

## Supplemental Material


Supplemental material - Death Rates of Elite and Professional American Athletes During Early Adulthood are Lower than the General Population
Supplemental material for Death Rates of Elite and Professional American Athletes During Early Adulthood are Lower than the General Population by John W. Orchard, Kimberley Harmon, Nicholas Orchard, Cindy J. Chang, Danica Sardelich, and Jessica J. Orchard in American Journal of Lifestyle Medicine
