# Supplementary material for: Death Rates of Elite and Professional American Athletes During Early Adulthood are Lower than the General Population
Source: Am J Lifestyle Med. 2026 Jan 26:15598276261418585. Online ahead of print. doi: 10.1177/15598276261418585 (PMC12846902; doi:10.1177/15598276261418585)
Supplement: Supplemental material - Death Rates of Elite and Professional American Athletes During Early Adulthood are Lower than the General Population [file sj-pdf-1-ajl-10.1177_15598276261418585.pdf]

Appendix

```
Select ?person ?personLabel ?dob ?sex ?dod ?cause ?personDescription

WHERE

{

?person wdt:P641 wd:Q847.#tennis sport (or other Q code for various other sports)

?person wdt:P27 wd:Q30; #USA nationality

       wdt:P21 ?sex;

wdt:P569 ?dob.

OPTIONAL { ?person wdt:P570 ?dod. } # Date of death (optional)

  OPTIONAL { ?person wdt:P509 ?cause. } # Date of death (optional)



 SERVICE wikibase:label { bd:serviceParam wikibase:language "[AUTO_LANGUAGE]".}

}
```

Wikidata sport IDs American-style football Q41323; Aquatic sports Q31920, Q7707 and Q7735; Athletics Q542; Auto racing Q5386; Baseball Q5369; Basketball Q5372; Boxing Q32112; Equestrian Q902378; Golf Q5377; Ice Hockey Q41466; Mixed martial arts Q114466; Mountaineering Q36908; Professional wrestling Q131359; Rowing Q159354; Soccer Q2736; Tennis Q847; Winter sports Q178131, Q186222 and Q38108.
